# Neuralgia

**Published:** 1881-05

**Authors:** 


HALL'S
### JOURNAL OF HEALTH
AND
MISCELLANY.

E. H. GIBBS, A. M., M. D. - - EDITOR.

Founded in 1854, and published Monthly without Interruption since that Time.

### NEURALGIA.

NEURALGIA means "nerve ache;" hence all pains are neuralgic, and indicate that there is undue pressure on the nerves of the part where the pain is located. Neuralgic affections of the head and face are, perhaps, the most distressing and the most persistent, as they are unfortunately, the most common. The object of this article is to refer briefly to some of the causes of such affections, which are usually preventable, and to some of the most approved methods of treatment. Persons who suffer half their time with these troubles, usually have no more than a vague and indefinite idea of their nature, but regard them as necessary evils to be heroically endured.

This seems to be an age of general investigation and improvement in everything except what relates to our physical well-being; as to that the vast majority of people seem to take no interest whatever; because when well they have other things to attend to, and when ill they are incapacitated for making the necessary investigations. There is no more mystery in regard to the causes and effects of disease than in the cause and effect of the derangement of any other complicated machinery. For example, a person goes from a warm room into the cold air. The sudden change of temperature checks the circulation of the blood in the small blood vessels, so that the heart pumps more blood into these vessels than can readily pass out, thus they become swollen, and this is called congestion. If this congestion occurs in the brain, and is slight, it produces headache; if severe, death often results. When congestion occurs in the blood vessels that pass through bone cavities, as the jaws or spine, the pressure on the nerve pulp causes severe, and often unremitting pain. The bone is unyielding and, except in toothache, it is difficult to procure direct and speedy relief.

It will be seen that the causes of these various troubles are simply mechanical; pressure on the nerves. The most important of all the means for avoiding these, and all other diseases and bodily discomforts, is to acquire a knowledge of the laws of health, and the penalties that follow their violation. The most frequent cause of neuralgic affections of the head and face is the diseased condition of the teeth. There is often a tendency to the rapid accumulation of tartar on the teeth. There are comparatively few people who are aware of the danger to health tnat neglect to remove these accumulations involves. When neglected, the tendency is to work down by additional accretion until it reaches the extremity of the fangs of the teeth, producing disease of the nerves at these points, which extends to the larger nerves. From this beginning is often developed one of the most painful diseases known to medical science. When this state of things is found to certainly exist, there is but one remedy, and when taken in time it is a radical cure. However sound and beautiful the teeth may seem to be, they should be removed, two or three at a time, until not one is left in the mouth. If the teeth have caused the trouble, the condition of the fangs of the first one that is extracted will prove it. There is always a cause for these diseases, as there is for all others, and the most important point is to find out the cause. No one need despair of finding the cause or the remedy.

Disease of the nerves of the face may commence in the nerves themselves, perhaps from cold, or the effect of malaria; but the remedy is the same when there is ulceration at the extremities of the fangs of the teeth. Sometimes the nerve which is incased in the jaw bone becomes so much diseased that it becomes necessary to saw into the channel and extract the diseased portion of the nerve. As a rule, decided and severe remedies must be resorted to in order to radically cure these affections when they have existed long enough to produce considerable disease of the nerves. There are remedies that often afford relief for a time in severe cases, and that even cure the milder forms of the disease.

---

CONUNDRUMS.—A new and characteristic anecdote of Carlyle: After having passed sleepless nights owing to the horrible noise made by a Cochin China cock in a neighboring garden, Carlyle interviewed the proprietor of the fowl, and expostulated. The owner, a woman, did not think Mr. Carlyle had much cause for complaint; the cock only crew three or four times in the night. "Eh, but woman," said the unfortunate philosopher, "if you only knew what I suffered waiting for him to crow?"

